# Green Tea Modulates Cytokine Expression in the Periodontium and Attenuates Alveolar Bone Resorption in Type 1 Diabetic Rats

**DOI:** 10.1371/journal.pone.0134784

**Published:** 2015-08-13

**Authors:** Gabriela Gennaro, Marcela Claudino, Tania Mary Cestari, Daniele Ceolin, Patrícia Germino, Gustavo Pompermaier Garlet, Gerson Francisco de Assis

**Affiliations:** Department of Biological Sciences, School of Dentistry of Bauru, São Paulo University, Bauru, São Paulo, Brazil; The University of Adelaide, AUSTRALIA

## Abstract

Diabetes mellitus comprises a heterogeneous group of disorders with the main feature of hyperglycemia. Chronic hyperglycemia increases the severity of periodontal disease via an exacerbated inflammatory response, activated by advanced glycation end products and their receptor, RAGE. Therefore, anti-inflammatory agents represent potential inhibitors of this pathological interaction. In particular, green tea has been shown to possess anti-inflammatory properties mediated by its polyphenol content. Objectives: This study investigated the mechanisms by which green tea attenuates the spontaneous onset of diabetes-induced periodontitis. Methods: Diabetes was induced in rats via a single intraperitoneal injection of streptozotocin (STZ). Diabetic and control animals were divided into water-treated and green tea-treated subgroups and were analyzed at 15, 30, 60 and 90 days after diabetes induction. Immunohistochemistry was performed to quantitatively evaluate tumor necrosis factor-α (TNF-α), receptor activator of nuclear factor kappa-B ligand (RANKL), osteoprotegerin (OPG), interleukin-10 (IL-10) and runt-related transcription factor 2 (RUNX-2) expression in serial sections of each hemimaxilla. Morphometric measurements of the distance from the cementum-enamel junction (CEJ) of the superior distal root of the first molar to the alveolar bone crest (ABC) were performed to assess bone loss. Results: Diabetes resulted in significant bone loss and alterations in the number of cells that stained positive for inflammatory mediators. In the diabetic rats treated with green tea, we observed a decreased number of cells expressing RANKL and TNF-α compared with that observed in the diabetic rats treated with water. Additionally, green tea increased the numbers of cells that stained positive for OPG, RUNX-2 and IL-10 in the diabetic rats. Conclusion: Green tea intake reduces expression of the pro-inflammatory cytokine TNF-α and the osteoclastogenic mediator RANKL to normal levels while increasing expression of the anti-inflammatory cytokine IL-10, the osteogenesis-related factor RUNX-2 and the anti-osteoclastogenic factor OPG. Therefore, green tea represents a potential therapeutic agent for the treatment of diabetes-related periodontal disease.

## Introduction

Diabetes mellitus represents a heterogeneous group of disorders affecting the metabolism of carbohydrates, lipids and proteins, with the main feature of hyperglycemia. The development of hyperglycemia has wide-ranging molecular and cellular effects, resulting in oxidative stress, upregulation of pro-inflammatory responses and vascular changes. Previous studies [1,2] have revealed that higher levels of inflammatory mediators, such as tumor necrosis factor alpha (TNF-α), interleukin-1β (IL-1β) and IL-6, are associated with classic diabetes complications, such as nephropathy, neuropathy, retinopathy and cardiovascular disease. Among the pathologies induced or exacerbated by diabetes, chronic hyperglycemia has been shown to affect the periodontal environment by increasing the prevalence and severity of periodontitis. Indeed, periodontal disease is considered the sixth complication of diabetes [3,4]. Several mechanisms have been proposed to explain the association between diabetes and periodontal disease. Diabetes might affect the periodontium via cytokine dysregulation, considering the destructive effects of pro-inflammatory mediators that have been linked to periodontal disease [5–8]. This hypothesis has been supported by studies revealing that poor glycemic control is significantly correlated with elevated expression of inflammatory mediators, osteoclastogenic factors and cytokines in gingival fluid [4,9,10]. Previous animal studies [11,12] have further demonstrated that a chronically hyperglycemic environment in periodontal tissue, even in the absence of external stimuli such as bacterial inoculation or silk ligatures, clearly results in the establishment and progression of periodontal disease. Together, these reports suggest that diabetes exacerbates the severity of periodontitis and potentially induces periodontal disease [11,12]. Moreover, diabetes-mediated inflammatory/immune dysregulation has been suggested to induce periodontitis development in response to commensal subgingival microflora [10,13,14].

In recent years, the health benefits of consuming green tea, including antidiabetic [15], anti-inflammatory [16], antiarthritic [17], antibacterial [18] and antioxidative [19] effects, have been investigated. Green tea, which is brewed from dried leaves of the plant *Camellia sinensis*, is among the most popular beverages worldwide. More than 80% of green tea polyphenols are catechins, which are derivatives of flavan-3-ol. The major catechins in green tea include (-)-epicatechin (EC), (-)-epicatechin gallate (ECG), (-)-epigallocatechin (EGC) and (-)-epigallocatechin 3-gallate (EGCG) [20,21].

EGCG has been reported to inhibit lipopolysaccharide (LPS)-induced inflammatory cytokine production *in vitro* [22]. In addition, Yang *et al*. [23] have examined the inhibitory effect of orally administered green tea polyphenols on LPS-induced TNF-α production, reporting that mice fed an extract containing these polyphenols exhibit decreased TNF-α production in response to intraperitoneal injection of LPS. Recently, the number of osteoclasts has been shown to be decreased in a dose-dependent manner following addition of green tea to culture medium containing mouse-derived bone marrow macrophages stimulated with receptor activator of nuclear factor kappa-B ligand (RANKL) and macrophage colony-stimulating factor (M-CSF) [24]. Furthermore, EGCG has been found to induce the apoptotic death of osteoclast-like multinucleated cells in a dose-dependent manner [25]. Although such pharmacological effects of catechins may be useful for the prophylaxis or treatment of inflammatory bone disease, few *in vivo* studies have confirmed the inhibitory effects of green tea on cytokine production.

Therefore, the purpose of this study was to investigate the effect of green tea on the spontaneous onset of diabetes-triggered periodontitis based on the quantitative and spatial evaluation of TNF-α, RANKL, IL-10, runt-related transcription factor 2 (RUNX-2), and osteoprotegerin (OPG) immunostaining patterns, as well as assessment of the oral microbiotic load, in the periodontal tissues of rats at 15, 30, 60 and 90 days after diabetes induction.

## Materials and Methods

### 2.1 Induction of diabetes and collection of samples

#### 2.1.1 Animals

Eighty 8-10-week-old adult male Wistar rats weighing approximately 250 g were used for these studies. The animals were housed in metabolic cages in groups of four animals per cage and were fed standard rat chow (Labina—Purina, São Paulo, Brazil) *ad libitum*. All of the experimental procedures performed on the animals were conducted with the approval of the Ethics Committee of the Bauru School of Dentistry of São Paulo State University (Protocol: CEEPA-014/2008).

#### 2.1.2 Experimental design

**Fig 1:**
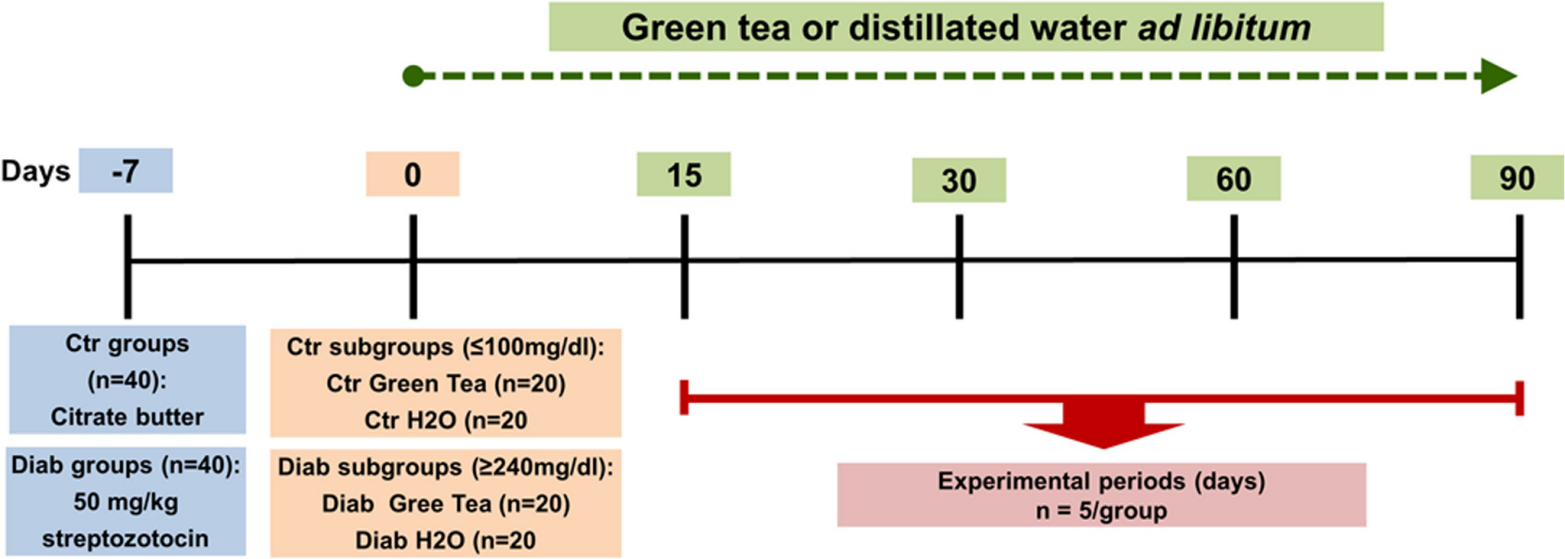
Experimental design and time schedules for the experimental subgroups and respective control subgroups. Diabetes was induced on day 0, and blood glucose was measured on day 7. STZ or citrate buffer dosing was initiated at 1 week before (day -7) the initial treatment with green tea or water (day 0), and histomorphometric and immunohistochemical analyses were performed on days 15, 30, 60 and 90.

Eighty animals were divided into the following two groups: type 1 diabetes mellitus (Diab, n = 40) and control (Ctr, n = 40). Each group was then divided into the following 4 subgroups: Diab Green Tea, Diab H2O, Ctr Green Tea and Ctr H2O (n = 20/subgroup). Experimental type 1 diabetes mellitus was induced in the rats in the Diab group via a single intraperitoneal injection of freshly prepared streptozotocin (STZ) (Sigma, St. Louis, MO, USA) at a dose of 50 mg/kg in 10 mM citrate buffer (pH 4.5) after overnight fasting. The glucose level in blood collected via a tail nick was measured using a glucometer (ACCU-CHEK Advantage meter system, Roche Diagnostics GmbH, Mannheim, Germany) at 7 days after injection of STZ. Rats displaying fasting blood glucose levels of above 240 mg/dL were considered diabetic and were included in this study. The animals in the Ctr group were injected with the same volume of citrate buffer. In addition, glucose levels were measured in the rats on the day of sacrifice. Regarding treatment allocation, the animals in the Diab Green Tea (n = 20) and Ctr Green Tea (n = 20) subgroups received green tea *ad libitum*, whereas those in the Diab H2O and Ctr H2O subgroups received distilled water. After 15, 30, 60 or 90 days, the animals (n = 5 per time point for each subgroup) were sacrificed via an overdose of ketamine and xylazine hydrochloride (Vetbrands Brazil Limited, Jacareí, SP), and the hemimaxillae were removed and fixed in 10% formalin solution (Fig 1).

### 2.2 Preparation of green tea

Green tea was provided in solution at a concentration of 7 g dried green tea leaves (imported from China by Santosflora Trade in Herbal Limited) per 1 L of hot water at an ideal temperature of 70°C. The leaves remained in the water for 10 min and were then leached. The solution was provided to the rats at room temperature, and it was replaced daily to preserve the beneficial properties of green tea.

### 2.3 Histological procedures

The hemimaxillae were decalcified by incubation in 4.13% EDTA and 1% formaldehyde (pH 7.2) at room temperature for 8 weeks. After rinsing with 0.1 M phosphate buffer, they were dehydrated with 90% ethanol followed by 100% ethanol, rinsed twice in xylene and embedded in molten paraffin at 56°C. Longitudinal semi-serial sections (5 μm thick) were generated and placed on silane-coated glass slides (Dako).

### 2.4 Histomorphometric evaluation of alveolar bone loss

In 3 histological sections per animal stained with hematoxylin and eosin, the distance from the alveolar bone crest (ABC) to the cementum-enamel junction (CEJ) of the distal root of the maxillary first molar was evaluated. Measurements were obtained from images of the coronal third of the distal root using a 40X immersion objective and digital analysis software (Axiovision 4.8).

### 2.5 Immunohistochemical procedures and determination of the number of immunolabeled cells per mm^2^ of tissue

Immunohistochemical staining was performed using the standard streptavidin-biotin peroxidase complex method. A goat polyclonal antibody against RANKL (sc-7628; Santa Cruz Biotechnology, Inc.) and rabbit polyclonal antibodies against IL-10 (250713; Abbiotec LLC, San Diego, CA, USA), OPG (ab73400; Abcam Biochemicals, USA), TNF-α (250844; Abbiotec, San Diego, CA, USA) and RUNX-2 (ab23981; Abcam Biochemicals, USA) were used. Following deparaffinization in xylene and rehydration in a decreasing ethanol gradient, endogenous peroxidase activity was blocked by soaking the tissue sections in 3% hydrogen peroxide for 20 min. After performing antigen retrieval by heating the sections in citrate buffer at 95°C for 20 min and blocking them in normal serum diluted in 7% fat-free milk for 40 min, the sections were incubated with primary antibodies against RANKL (1:75), OPG (1:150), TNF-α (1:50), IL-10 (1:50) and RUNX-2 (1:75) for 2 h at room temperature. After washing in PBS, the sections were incubated with a biotinylated secondary antibody (sc-2774 or sc-2040; Santa Cruz Biotechnology, Inc.) for 1 h, followed by incubation with streptavidin-peroxidase complex (DakoCytomation, Glostrup, Denmark) for 20 min. The immune complexes were visualized by incubating the sections in 3–3’-diaminobenzidine (DakoCytomation, Glostrup, Denmark) for 5 min. Then, the immunostained sections were counterstained with hematoxylin. The negative control sections were incubated in 3% bovine serum albumin in PBS instead of the primary antibody, and they did not display any specific immunoreactivity.

All of the immunolabeled sections were assigned a random numerical sequence to codify the experimental periods and groups throughout the analysis. A single trained investigator analyzed the sections and quantified the number of immunolabeled cells per mm^2^ of tissue. For each hemimaxilla, 15 microscopic fields (5 fields/histological section) of the periodontal ligament (PDL) surrounding the distal root of the first molar were examined. Images were captured using a high-resolution digital camera (AXIOCAM HRc; Carl Zeiss) attached to an Axioscope 2 microscope (Carl Zeiss) equipped with a 100X oil immersion objective lens. Immunostained cells were counted using digital analysis software (Axiovision 4.8), and the results are presented as the number of immunolabeled cells per mm^2^ of tissue for each animal.

### 2.6 DNA extraction and real-time PCR

For detection of the periodontopathogens *Porphyromonas gingivalis*, *Prevotella nigrescens*, *Tannerella forsythia*, *Treponema denticola* and *Aggregatibacter actinomycetemcomitans* in the biofilm and host tissue, bacterial DNA was extracted from a sample of periodontal support tissue (alveolar bone, PDL and cementum). Periodontal support tissue samples were frozen in liquid nitrogen and mechanically fragmented and homogenized in sterile Milli-Q water. DNA was extracted from these samples via sequential phenol chloroform extraction and precipitation using a salt/ethanol solution. Bacterial load (16S) was quantified using a RealTime PCR l DNA purification system (Promega) as previously described [26]. Real-time PCR analyses were performed using a MiniOpticon system (Bio-Rad, Hercules, CA, USA), SYBR Green Master Mix (Invitrogen), specific primers at a concentration of 100 nM (Table 1) and 5 ng of DNA for each reaction, as previously described [26,27].

**Table 1:** 

Target	*Sense and antisense sequences*	*At (°C)*	*At (°C)*	*At (°C)*
**16S**	***CGCTAGTAATCGTGGATCAGAATGTGTGACGGGCGGTGTGTA***	***60***	***72***	***69***
**P. gingivalis**	***TACCCATCGTCGCCTTGGT/CGGACTAAAACCGCATACACTTG***	***60***	***84***	***126***
**T. denticola**	***AGAGCAAGCTCTCCCTTACCGT/TAAGGGCGGCTTGAAATAATGA***	***59***	***80***	***105***
**T. forsythia**	***GGGTGAGTAACGCGTATGTAACCT/ACCCATCCGCAACCAATAAA***	***59***	***79***	***127***
**A. actinomycetemcomitans**	***ATGCCAACTTGACGTTAAAT/AAACCCATCTCTGAGTTCTTCTTC***	***60***	***78***	***557***

Abbreviations: At, annealing temperature; bp, amplicon size in base pairs; Mt, melting temperature.

### 2.7 Statistical analyses

Two-way analysis of variance (ANOVA) was conducted to analyze data for all of the groups. Tukey’s test was then performed to detect significant differences between the groups related to time. The significance level was set at *P <* 0.05, and all calculations were performed using GraphPad Prism 5.0 software (GraphPad Software Inc., USA).

## Results

### 3.1 Clinical data

**Fig 2:**
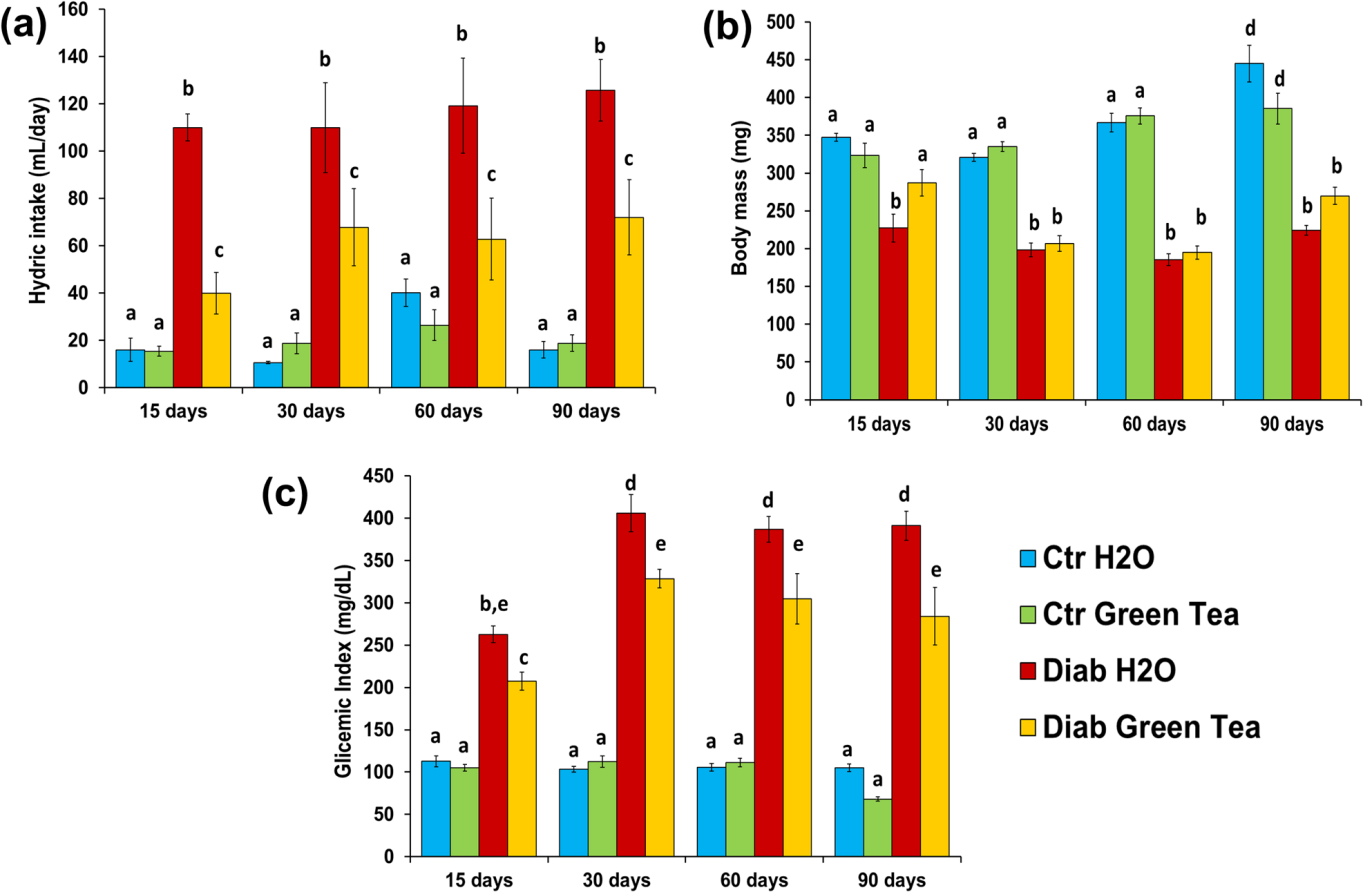
Clinical data for diabetic and normoglycemic rats for the entire experimental period. a) The diabetic rats exhibited greater fluid intake than the non-diabetic rats throughout the experimental period. b) The control group exhibited a higher body weight than the diabetic group (means of 362.4± 15.0 and 222.9±9.5, respectively) throughout the experimental period. c) The glycemic index was within the normal range in the control group, but the diabetic group exhibited a glycemic index that was compatible with diabetes. Two-way ANOVA followed by Tukey’s test (different letters = p<0.05); n = 5 animals/group.

Fluid intake (Fig 2A) and body weight (Fig 2B) were measured until the day of euthanasia. Blood was collected from the tail vein of fasted (12–14 h) rats for measurement of blood glucose levels (Fig 2C) using a glucometer (Accu-Check Advantage meter system, Roche Diagnostics GmbH, Mannheim, Germany). The rats in the Ctr Green Tea and Ctr H2O subgroups consumed a mean of 20.3±3.9 mL/day (p = 1), whereas those in the Diab Green Tea and Diab H2O subgroups consumed 60.7±5.5 mL/day (p<0.0001) and 116.3±3.0 mL/day (p<0.0001), respectively. The body weights of the animals was similar between the Ctr and Diab Green Tea subgroups at 15 days (mean of 315.7±13.1 g), whereas that of the animals in the Diab H2O subgroup was 28% lower (227.2±41.6 g) than that of the animals in the Ctr H2O subgroup. Body weight increased in the Ctr group (to a mean of 415.3±18.6 g at 90 days) but remained constant in the Diab group (at a mean of 247.1±8.9 g) throughout the experimental period. The mean glucose levels were 102.9±6.1 mg/dL, 281.2±21.4 mg/dL and 361.7±16.0 mg/dL for the Ctr group, Diab Green Tea subgroup and Diab H2O subgroup, respectively, during the entire experimental period.

### 3.2 Histomorphometric analysis

**Fig 3:**
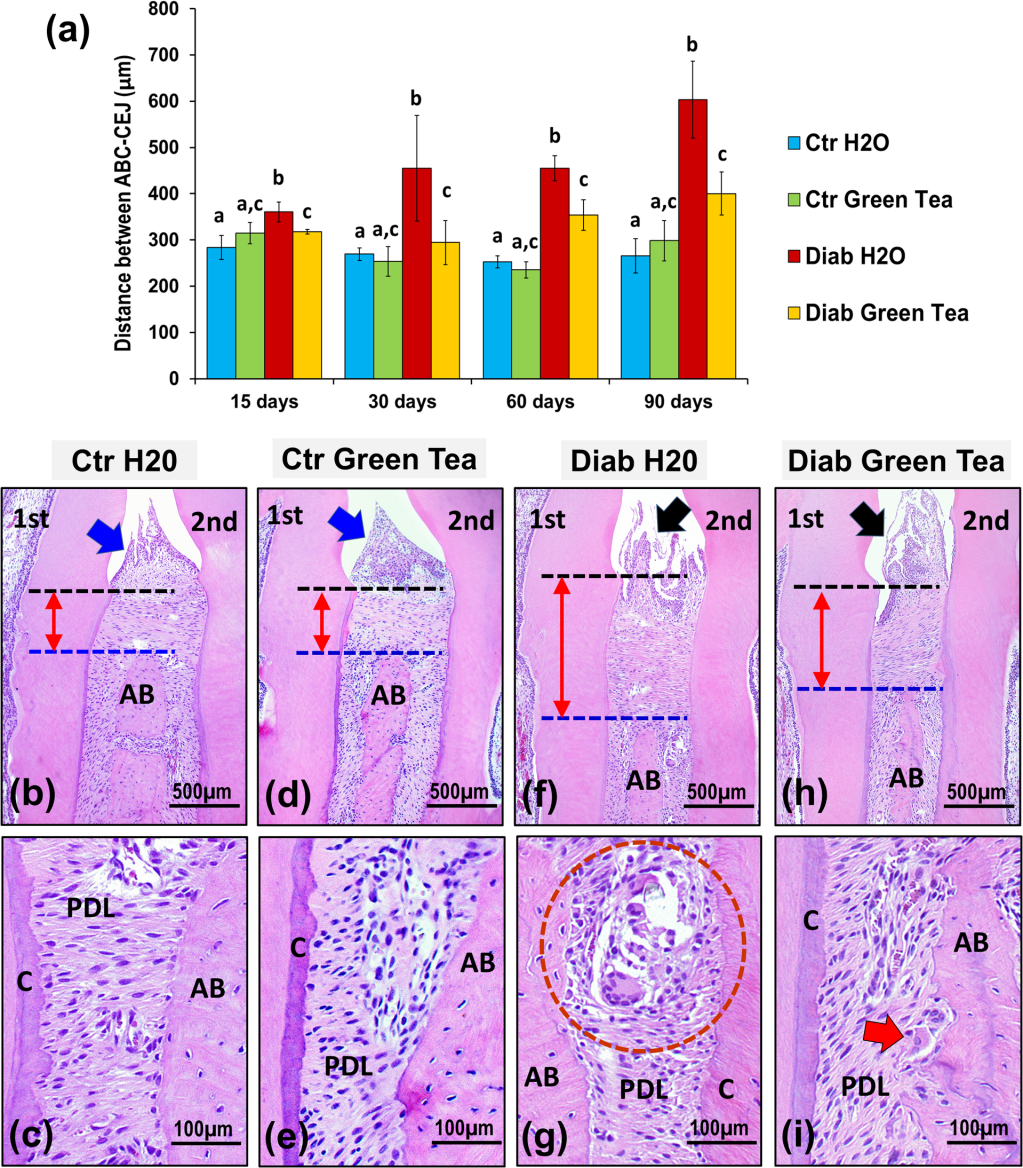
Distance (double arrow) between the ABC (black dotted line) and CEJ (blue dotted line) of the first molar distal root (a) and photomicrographs of the interdental space between the 1^st^ and 2^nd^ maxillary molars in the control group (b-e) and diabetic group (f-i) at 90 days. In the control group, the mean ABC-CEJ distance was 271.6±25.7 μm, demonstrating that the integrity of the junctional epithelium (blue arrows) and the alveolar bone (AB) was maintained. In the Diab H2O subgroup, the ABC-CEJ distance was longer than that in the other groups due to junctional epithelium extension (black arrow) and extensive bone loss (double arrow), corresponding to severe inflammation and enhanced osteoclastic activity (red circle). In the Diab Green Tea subgroup, the increases in the ABC-CEJ distance (double arrow) and bone loss (AB) were smaller than those in the Diab H2O subgroup, demonstrating the alleviation of inflammation and osteoclastic activity (red arrow). In the graph: two-way AVONA followed by Tukey’s test (different letters = p<0.05), n = 5 animals/group. In the photomicrographs: PDL = periodontal ligament; c = cementum; HE; 10X and 40X objectives.

Morphometric analysis (Fig 3A) demonstrated that the ABC-CEJ distance in the Ctr group was shorter than that in the Diab group throughout the entire experimental period (means of 271.6±25.7 μm and 405.0±41.5 μm, respectively). In the Diab group, the rats that received water exhibited more extensive bone loss than those that received green tea throughout the experimental period (ABC-CEJ distances of 468.4±61.4 μm and 341.5±26.1 μm, respectively). In accordance with these morphometric results, the histological images demonstrated that the bone crest and junctional epithelium remained at normal levels in the Ctr group at 90 days (Fig 3B–3E) but that alveolar bone resorption was extensive and that the junctional epithelium was lengthened in the Diab group, especially in the Diab H2O subgroup (Fig 3F–3I).

### 3.3 Immunohistochemical analysis

**Fig 4:**
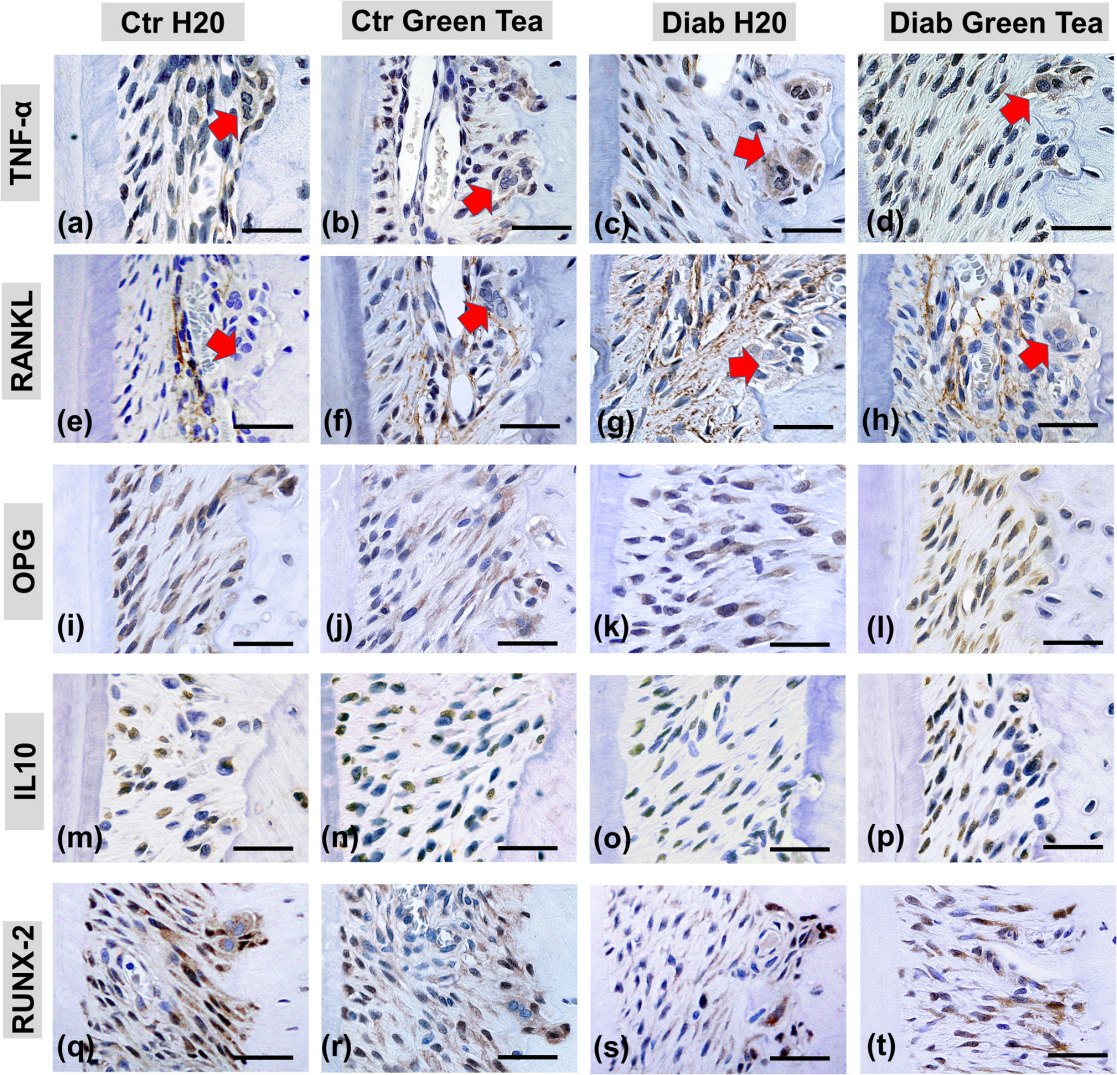
Photomicrographs of cells immunostained for the pro-inflammatory cytokines TNF-α and RANKL, the anti-inflammatory cytokines OPG and IL-10 and the transcriptional activator of osteoblast differentiation RUNX-2 at 90 days. a-h) The Diab H2O rats exhibited more immunostaining for the pro-inflammatory cytokines TNF-α and RANKL. Immunostaining is evident around osteoclastic cells (red arrow), showing their intensive resorptive activities. i-p) The rats in the Diab H2O subgroup exhibited markedly fewer immunostained cells for anti-inflammatory cytokines than those in the other groups. q-t) The number of cells that stained positive for RUNX-2 was markedly lower in the Diab H2O subgroup than in the other subgroups. In the photomicrographs: 15 microscopic fields (5 fields/histological section) of the periodontal ligament surrounding the distal root of the first molar were examined. Immunohistochemical staining was performed using the standard streptavidin-biotin peroxidase complex method with a 100X objective.

**Fig 5:**
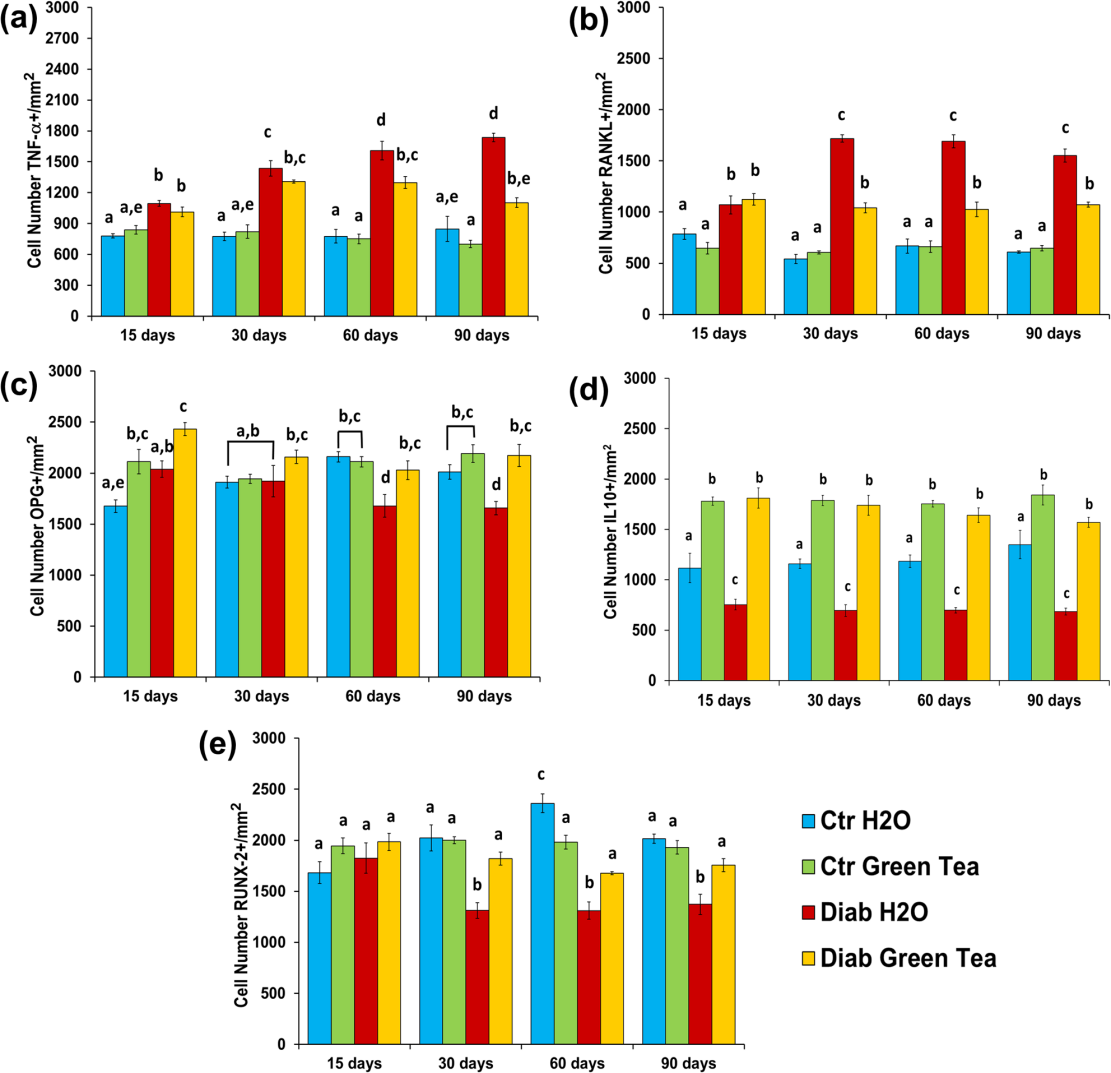
Number of cells immunostained for the pro-inflammatory cytokines TNF-α (a) and RANKL (b), the anti-inflammatory cytokines OPG (c) and IL-10 (d) and the transcriptional activator of osteoblast differentiation RUNX-2 (e). In the control group, the number of cells that stained positive for the pro-inflammatory cytokines TNF-α and RANKL was significantly lower than that in the diabetic group (715.1±107.3 cells/mm^2^ and 1305±111 cells/mm^2^ for the control and diabetic groups, respectively). However, in the Diab Green Tea subgroup, the number of cells that stained positive for both pro-inflammatory cytokines was significantly lower than that in the Diab H2O subgroup at 30 and 90 days (1141±71 cells/mm^2^ and 1653±104 cells/mm^2^ for the Diab Green Tea and Diab H2O subgroups, respectively). Furthermore, the number of cells that stained positive for the anti-inflammatory cytokines and RUNX-2 was significantly lower in the Diab H2O subgroup than in the other subgroups. In contrast, in the Diab Green Tea subgroup, the number of cells that stained positive for OPG and RUNX-2 was similar and the number of cells that stained positive for IL-10 was increased compared with those in the Ctr H2O subgroup at 30, 60 and 90 days of treatment, suggesting that green tea has a beneficial role in the reduction of periodontal disease in diabetic rats. In the graph: two-way ANOVA followed by Tukey’s test (different letters = p<0.05); n = 5 animals/group.

Immunoreactive cells were detected in 5 sequential regions of periodontal tissue that included the coronal third of the distal root of the superior first molar, as illustrated in Fig 4. First, we evaluated the number of cells that stained positively for pro-inflammatory cytokines associated with bone resorption, such as TNF-α and RANKL. We detected an increased number of TNF-α-positive cells (Fig 4A–4D and Fig 5A) in both Diab subgroups compared with the Ctr subgroups at all time points examined. We observed an increase in immunoreactive cells in the Diab H2O subgroup between 30 and 90 days (1434.8±76.1 cells/mm^2^ and 1737.4±41.5 cells/mm^2^, respectively). However, the number of immunoreactive cells in the Diab Green Tea subgroup (mean of 1236.2±42.37 cells/mm^2^) remained constant and was significantly lower than that in the Diab H2O subgroup at 60 and 90 days.

We found statistically significant increases in the number of RANKL-positive cells (Fig 4E–4H and Fig 5B) in the Diab subgroups compared with the number of cells in the respective Ctr subgroups at all time points examined. Comparison of the Diab Green Tea and Diab H2O subgroups revealed that the animals that received green tea clearly displayed significantly fewer RANKL-positive cells (mean of 1046.7±31.6 cells/mm^2^) than the diabetic animals that received water (mean of 1653.6±41.9 cells/mm^2^) at 30, 60 and 90 days after diabetes induction.

Next, we evaluated the number of cells that stained positively for anti-inflammatory cytokines, such as OPG and IL-10. The diabetic rats that ingested green tea displayed a greater number of OPG-positive cells (2211.5±74.6 cells/mm^2^) than those that received water (1791.6±78.3 cells/mm^2^) (Fig 4I–4L and Fig 5C), and this difference was statistically significant at 15, 60 and 90 days. Evaluation of the number of IL-10-positive cells (Fig 4M–4P and Fig 5D) showed that more immunoreactive cells were present in the rats treated with green tea (independent of glycemic status) than in those treated with water at all time points (green tea subgroups: 1740.9±38.0 cells/mm^2^; H2O subgroups: 955.1±114.4 cells/mm^2^).

To detect possible changes in the bone cellular differentiation rate caused by diabetes, we evaluated the number of RUNX-2-positive cells via immunostaining (Fig 4Q–4T and Fig 5E). The Diab subgroup that received water displayed fewer RUNX-2-positive cells (mean of 1331.8±12.9 cells/mm^2^) than the other subgroups (mean of 1951.8±83.2 cells/mm^2^) at 30, 60 and 90 days. Moreover, the Diab Green Tea subgroup (mean of 1809.7±50.2 cells/mm^2^) displayed a similar number of RUNX-2-positive cells compared with the Ctr subgroups (mean of 1992.4±77.5 cells/mm^2^) throughout the experimental period.

### 3.4 Detection of periodontopathogens via real-time PCR

We also evaluated the overall microbial load in the oral cavity, and the results demonstrated that the levels of 16S bacterial DNA were similar between the diabetic and control groups at all time points evaluated. DNA samples extracted from periodontal tissue sections were investigated for the presence of classic periodontal pathogens (*A*. *actinomycetemcomitans*, *P*. *gingivalis*, *Prevotella nigrescens*, *T*. *forsythia* and *T*. *denticola*); however, none of the samples tested positive at any time point in the Diab group or the Ctr group.

## Discussion

Periodontal disease is more severe and frequent in the presence of diabetes [3,4,10] because of increased levels of pro-inflammatory cytokines and osteoclastogenic factors [7]. Whereas modulation of established periodontal disease by diabetes appears to be the consensus in the scientific literature, the recently demonstrated co-induction of periodontal disease by diabetes [3,28] is less commonly reported. In this context, the co-induction hypothesis is characterized by a “two-hit model,” in which the “first hit” is hypothetically provided by the oral microflora, and the “second hit” is caused by an exacerbated inflammatory response induced by systemic diseases, such as diabetes [28]. In accordance with this co-induction hypothesis, our results demonstrated that diabetes induction triggered alterations that were typical of periodontal disease, even in the absence of periodontitis [11,12]. We investigated the overall bacterial load in the oral cavity, as represented by the 16S bacterial DNA level. However, our results did not reveal any variation in the periodontal bacterial load, suggesting the absence of quantitative differences in biofilm formation between the two groups. Additionally, we investigated the presence of classical periodontal pathogens, including *A*. *actinomycetemcomitans*, *P*. *gingivalis*, *P*. *nigrescens*, *T*. *forsythia* and *T*. *denticola*; however, none of the samples tested positive in the diabetic group or control group throughout the experimental period. Taken together, the results of a previous study [12,29] and our data suggest that the presence of diabetes does not affect the qualitative or quantitative profile of dental biofilms. These findings indicate that diabetes affects the expression of mediators related to the establishment and progression of periodontal disease and that these changes do not appear to be accompanied by a marked change in the overall bacterial load in the oral cavity. Therefore, factors other than the oral microflora may act as inducers of periodontal disease, representing the “second hit,” in distinct areas of periodontal tissue.

In addition, we observed less bone loss at all experimental time points in the diabetic rats that ingested green tea compared with those that ingested water. This result can be explained by the reduced glycemic index in the diabetic rats that consumed green tea *ad libitum* throughout the experimental period. Although the daily consumption of green tea did not completely protect against STZ-induced hyperglycemia and bone loss in the experimental rats, the findings of this study suggest that daily green tea ingestion may represent a useful therapeutic strategy for modulation of the negative effects of diabetes on periodontal disease. With respect to diabetes, other animal studies have shown that administration of green tea to STZ-induced diabetic rats reduces the blood glucose level, promotes hypolipidemic responses, enhances antioxidative activity, improves kidney function, and protects cardiac function [30]. Furthermore, a recent review has suggested that tea and its bioactive components might decrease the risk of fracture by improving bone mineral density (BMD) and promoting osteoblastic activities while suppressing osteoclastic activities [31,32]. Shimazaki et al. have suggested that the antioxidants present in green tea decrease systemic inflammation, thereby reducing periodontal tissue loss [33].

We next investigated the possible effects of diabetes on the host immune response by performing immunohistochemical analysis. In particular, we observed a greater number of cells that stained positively for TNF-α in the diabetic group than in the control group at all time points examined. In accordance with our data, higher serum levels of this cytokine have been reported in patients with poorly controlled diabetes compared with healthy individuals [34]. Additionally, macrophages from diabetic patients have been shown to possess an enhanced capacity for producing TNF-α compared with macrophages from non-diabetic individuals [35]. In fact, in our previous study, increased expression of TNF-α was observed in the rat periodontium after diabetes induction [12]. In the current study, comparison of water treatment to green tea treatment in diabetic rats revealed that the green tea-treated subgroup had fewer TNF-α-positive cells than the water-treated subgroup at 60 and 90 days after diabetes induction. Similarly, previous studies of mice have shown that green tea polyphenols administered via oral gavage at 2 h prior to intraperitoneal injection of 40 mg of LPS lead to a dose-dependent decrease in LPS-induced TNF-α production in serum. Moreover, green tea polyphenols have been shown to completely inhibit LPS-induced lethality in mice [23].

It has been well established that inflammatory mediators, such as TNF-α, are positive regulators of osteoclastogenesis [36]. In periodontal lesions, the balance between the expression of anti-inflammatory and pro-inflammatory mediators has been hypothesized to impact the outcome of periodontal disease, possibly by regulating the balance between RANKL and OPG [7,36,37]. We found an increased number of RANKL-positive cells at all time points after diabetes induction, independent of water or green tea intake. However, the diabetic rats treated with water showed an increased number of cells with positive RANKL staining compared with rats treated with green tea at 30, 60 and 90 days. Moreover, the production of RANKL has been found to be suppressed in osteoblasts infected with *Staphylococcus aureus* after treatment with green tea-derived catechins, indicating that green tea has an anti-inflammatory effect [38]. The addition of green tea to culture medium containing macrophages cultured with RANKL and M-CSF has also been shown to decrease osteoclast formation [21].

In contrast to RANKL, OPG is a naturally occurring decoy receptor that inhibits osteoclast formation and bone resorption. Thus, the effects of RANKL are counteracted by those of OPG, which strongly inhibits the RANK–RANKL interaction [39]. Moreover, an imbalance in this system is a pivotal component of the etiology of certain bone disorders [7]. According to the discussion presented above, in periodontal disease, the RANKL level is increased in diseased periodontal tissues, and the balance between RANKL and OPG expression has been hypothesized to determine periodontal disease severity [5,7,8]. We observed a decreased number of OPG-positive cells in diabetic rats at the late stages of disease (60 and 90 days). However, green tea exerted a protective anti-inflammatory effect over time, as the diabetic green tea-treated group displayed an increased number of OPG-positive cells compared with the diabetic water-treated group. EGCG did not exert any effect on OPG synthesis by osteoblast-like cells [40] and did not alter the expression of OPG in osteoblasts cultured in the presence of osteoclastogenic factors [41]. These differences may have arisen because our experiment was performed *in vivo* instead of *in vitro*. Furthermore, the rats in our study received whole green tea rather than specific isolated catechins, and catechins aside from EGCG may exert anti-inflammatory effects that result in a decrease in the RANKL level and an increase in the OPG level, as observed in our study.

Because the alveolar bone loss exhibited by diabetic rats appears to be spontaneous and irreversible, diabetes likely affects bone formation. Thus, we investigated the number of cells that stained positively for RUNX-2, a transcription factor that is considered to be a critical regulator during differentiation of the osteoblast linage [42]. The diabetic rats displayed fewer RUNX-2-positive cells than the control rats at 30 days after diabetes induction. Indeed, bone formation has been previously shown to be significantly reduced in diabetic mice due to decreased expression of RUNX-2 [40]. In a recent study, diabetic mice have been shown to exhibit greater orthodontic tooth movement in association with the increased expression of osteoclastic factors and decreased expression of osteoblastic markers, including RUNX-2 [43]. Taken together, these results suggest that diabetes may lead to an imbalance in bone formation processes due to defects in cellular differentiation resulting from the decreased expression of transcription factors such as RUNX-2. However, the diabetic rats treated with green tea displayed a higher number of RUNX-2-positive cells than those treated with water. Similarly, in a murine bone marrow mesenchymal stem cell line, the mRNA expression levels of RUNX-2, osterix, osteocalcin and alkaline phosphatase have been reported to be increased after 48 h of EGCG treatment [20].

Periodontitis, bone resorption and diabetes appear to be closely related, and chronic inflammation is the common factor among these conditions. In particular, the circulating pro-inflammatory cytokines that are elevated in diabetes may promote osteoclast activity and bone resorption by modulating the RANK/RANKL/OPG pathway [44]. In addition, IL-10 counteracts inflammatory bone loss by downregulating the signaling and activity of pro-inflammatory cytokines [45]. Indeed, IL-10 has been demonstrated to confer protection against tissue destruction by inhibiting the RANK system and by stimulating the production of OPG, which prevents the RANK–RANKL interaction [8,45].

Altered B cell functioning in diabetes mellitus patients leads to increased inflammation via the following two mechanisms: increased pro-inflammatory IL-8 production and a lack of anti-inflammatory/protective IL-10 production [46]. In our study, the diabetic water-treated subgroup displayed fewer IL-10-positive cells than the non-diabetic group at all time points examined. Similarly, IL-10 gene expression has been shown to be significantly reduced in the bone cells of diabetic rats [47].

However, the diabetic rats that ingested green tea displayed more IL-10-positive cells than those that ingested water, beginning at the initial time point examined. In accordance with our results, EGCG, the primary polyphenolic component of green tea, has been shown to significantly elevate the level of the circulating anti-inflammatory cytokine IL-10 and to effectively delay the onset of diabetes in non-obese diabetic mice [48]. Interestingly, all rats that consumed green tea displayed an increased number of IL-10-positive cells. Thus, we observed similar IL-10 levels between the control rats and the diabetic rats treated with green tea.

**Fig 6:**
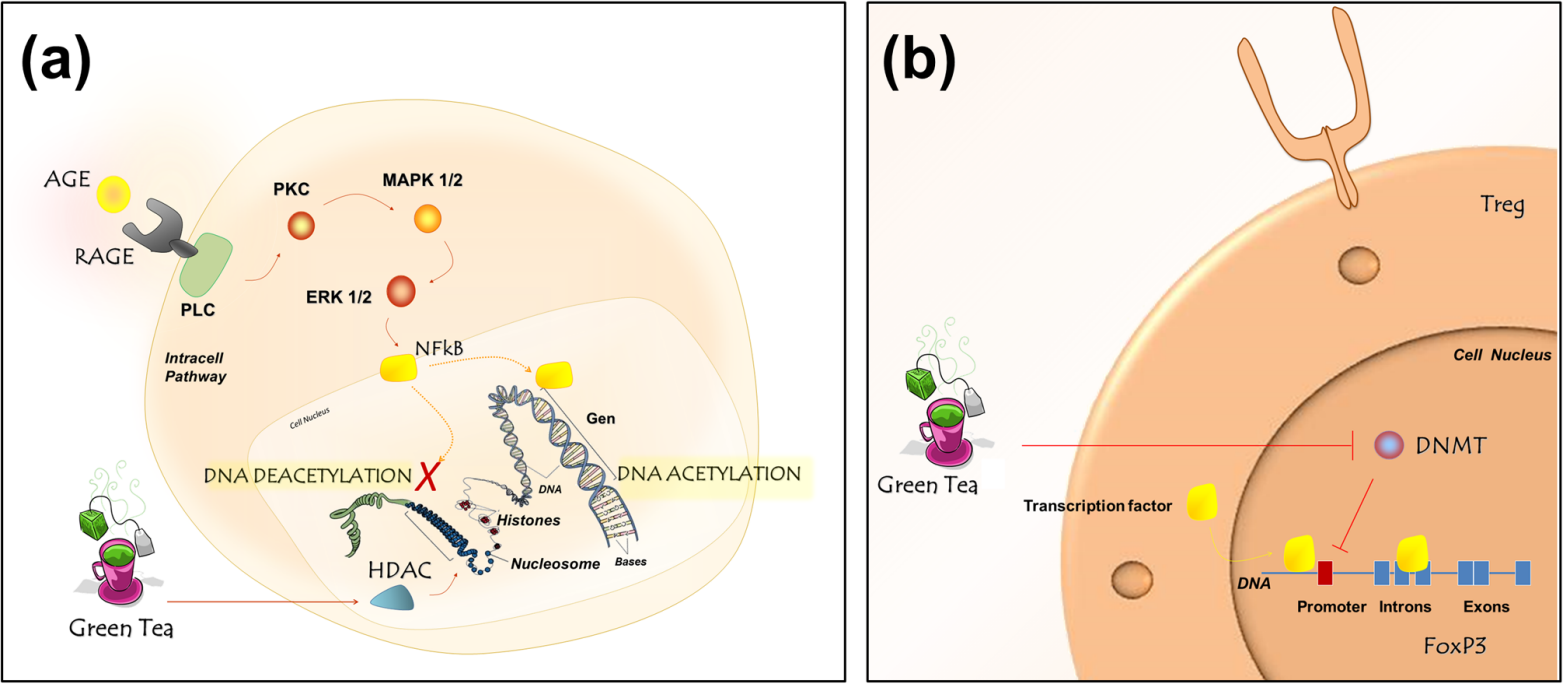
Schematic of potential cellular pathways mediating the effects of green tea. a) Green tea catechins may alter gene expression by inhibiting DNMT activity, resulting in reactivation of methylation-silenced genes such as Foxp3 and increased IL-10 expression. b) Catechins may stimulate upregulation of HDAC activity, thereby repressing gene transcription by promoting DNA winding, limiting the accessibility of certain DNA regions to the transcription factor NF-ĸB.

Finally, the potential mechanisms underlying the anti-inflammatory effects of green tea should be discussed. Regulatory T cells (Tregs) play a pivotal role in IL-10 expression and in protective anti-inflammatory immune responses. Treg differentiation is regulated by the transcription factor Foxp3, as demethylation of the Foxp3 promoter in naïve CD4^+^ T cells using DNA methyltransferase (DNMT) inhibitors has been shown to result in the de-repression and stable expression of Foxp3 and in the subsequent differentiation of naïve CD4^+^ cells into Tregs [49]. Recent studies have further indicated that EGCG alters gene expression by inhibiting DNMT activity, resulting in reactivation of methylation-silenced genes and, consequently, IL-10 expression [50,51] (Fig 6A).

Another pathway that may be involved in the anti-inflammatory effects of green tea is histone acetylation–deacetylation, which is an epigenetic event that plays an important role in inflammation. Acetylation at specific lysine residues in the N-terminal tail of core histones by histone acetyltransferases results in the uncoiling of DNA and an increase in the accessibility of certain DNA regions to transcription factors [52]. Furthermore, histone deacetylation by histone deacetylases (HDACs) represses gene transcription by promoting DNA winding, thereby limiting the access of certain DNA regions to transcription factors. As a result, EGCG alters HDAC activity and strongly suppresses NF-kB [53] (Fig 6B).

Taken together, our results demonstrate that green tea intake reduces the expression of the pro-inflammatory cytokine TNF-α and the osteoclastogenic mediator RANKL to normal levels and increases the expression of the anti-inflammatory cytokine IL-10, the osteogenesis-related factor RUNX2 and the anti-osteoclastogenic factor OPG. Furthermore, green tea appears to represent a potential therapeutic agent for the treatment of diabetes-related periodontal disease due to its action on inflammatory pathways. However, there is a large amount of inter-individual variation in green tea intake due to differences in the glycemic index. Therefore, additional studies are needed to further investigate the effects of green tea according to the amount consumed.
